# Guidance of treatment decisions in risk-adapted primary radiotherapy for prostate cancer using multiparametric magnetic resonance imaging: a single center experience

**DOI:** 10.1186/s13014-015-0338-3

**Published:** 2015-02-22

**Authors:** Cedric Panje, Thierry Panje, Paul Martin Putora, Suk-kyum Kim, Sarah Haile, Daniel M Aebersold, Ludwig Plasswilm

**Affiliations:** Department of Radiation Oncology, Kantonsspital St. Gallen, St. Gallen, Switzerland; Department of Radiology and Nuclear Medicine, Kantonsspital St. Gallen, St. Gallen, Switzerland; Clinical Trials Unit, Kantonsspital St. Gallen, St. Gallen, Switzerland; Department of Radiation Oncology, Bern University Hospital, Bern, Switzerland

**Keywords:** MRI, Multiparametric, Prostate cancer, Radiotherapy

## Abstract

**Background:**

Magnetic resonance imaging (MRI) of the prostate is considered to be the most precise noninvasive staging modality for localized prostate cancer. Multiparametric MRI (mpMRI) dynamic sequences have recently been shown to further increase the accuracy of staging relative to morphological imaging alone. Correct radiological staging, particularly the detection of extraprostatic disease extension, is of paramount importance for target volume definition and dose prescription in highly-conformal curative radiotherapy (RT); in addition, it may affect the risk-adapted duration of additional antihormonal therapy. The purpose of our study was to analyze the impact of mpMRI-based tumor staging in patients undergoing primary RT for prostate cancer.

**Methods:**

A total of 122 patients admitted for primary RT for prostate cancer were retrospectively analyzed regarding initial clinical and computed tomography-based staging in comparison with mpMRI staging. Both tumor stage shifts and overall risk group shifts, including prostate-specific antigen (PSA) level and the Gleason score, were assessed. Potential risk factors for upstaging were tested in a multivariate analysis. Finally, the impact of mpMRI-based staging shift on prostate RT and antihormonal therapy was evaluated.

**Results:**

Overall, tumor stage shift occurred in 55.7% of patients after mpMRI. Upstaging was most prominent in patients showing high-risk serum PSA levels (73%), but was also substantial in patients presenting with low-risk PSA levels (50%) and low-risk Gleason scores (45.2%). Risk group changes occurred in 28.7% of the patients with consequent treatment adaptations regarding target volume delineation and duration of androgen deprivation therapy. High PSA levels were found to be a significant risk factor for tumor upstaging and newly diagnosed seminal vesicle infiltration assessed using mpMRI.

**Conclusions:**

Our findings suggest that mpMRI of the prostate leads to substantial tumor upstaging, and can considerably affect treatment decisions in all patient groups undergoing risk-adapted curative RT for prostate cancer.

## Background

External beam radiotherapy (RT) of the prostate has been established as an effective therapeutic option for localized prostate cancer as a single treatment modality or in conjunction with systemic androgen deprivation therapy (ADT); it has achieved excellent rates of locoregional and biochemical control [1-3]. Recent phase III studies have demonstrated an additional improvement in oncological outcome for patients presenting with adverse risk factors including advanced T-stage, markedly elevated serum prostate-specific antigen (PSA) and high-grade disease by means of treatment intensification such as radiation dose escalation and the addition of ADT [4-6]. Current consensus guidelines recommend extension of the target volume beyond the prostatic capsule for locally advanced disease (T3 stage) to account for extracapsular extension (ECE) and seminal vesicle invasion (SVI) [7]. Such an accurate target volume definition is particularly important when highly conformal techniques such as image-guided intensity-modulated radiotherapy (IMRT) are used [8]. However, as a definite pathological specimen is not available like after radical prostatectomy, curative RT for prostate cancer relies primarily on accurate clinical and radiological tumor staging for risk group-adapted treatment intensification as well as for target volume delineation [7,9].

To date, magnetic resonance imaging (MRI) of the prostate is considered to be the most accurate imaging modality available for the noninvasive determination of the local extent of prostate cancer [10,11]. More recently, prostate morphological imaging involving T1- and T2-weighted MRI has been routinely complemented by multiparametric dynamic sequences such as diffusion-weighted imaging (DWI) and dynamic contrast-enhanced imaging, which have been shown to further increase specificity and sensitivity [12-14]. MRI of prostate cancer allows for the reliable detection of adverse pathological features such as ECE and SVI [15-18]. It has also shown a superior congruence with the final surgical-pathological staging relative to digital rectal examination, transrectal ultrasound-guided biopsy and computed tomography (CT) [19,20], as well as compared with prediction tools such as the Partin tables [21,22] and the Kattan nomogram [23].

A small number of previous studies have demonstrated that morphological MRIs of localized prostate cancer resulted in a significant tumor stage shift with consequent implications regarding target volume definition [24] and more accurate prediction of treatment outcome [25]. However, to our knowledge, the impact of state-of-the-art multiparametric MRI (mpMRI) of the prostate on curative RT for prostate cancer and additional ADT has not yet been specifically investigated. Consequently, the purpose of our study was to retrospectively analyze the value of mpMRI for prostate cancer staging before curative RT and its impact on treatment decisions.

## Methods

### Patient selection

After review and the approval of the institutional ethics committee (Ethics Committee St. Gallen, Switzerland), 160 patients with clinically localized prostate cancer were identified. These patients had been referred to the Cantonal Hospital St. Gallen between January 2010 and December 2013 for primary RT, based on patient preference or medical inoperability; they had all received a mpMRI of the prostate before RT. For further analysis patients who met the following staging criteria before undergoing a pelvic MRI scan (n = 122) were included: histopathological confirmation of prostate cancer using transrectal ultrasound-guided biopsy evaluated according to the Gleason grading system [26]; a complete medical history and physical examination; a serum PSA measurement; and a CT scan of the abdomen and pelvis. Clinical stage before the MRI scan was determined using the 2010 International Union Against Cancer (UICC), 7th edition staging criteria [27].

Based on the pre-treatment serum PSA, histopathological Gleason Score and clinical staging patients were assigned to low-risk, intermediate-risk and high-risk groups according to the 2014 National Comprehensive Cancer Network (NCCN) guidelines on prostate cancer (Table 1) [28].

**Table 1:** **Risk group classification for localized prostate cancer according to the National Comprehensive Cancer Network guidelines**

	**T Stage**	**PSA (ng/ml)**	**Gleason score**
**(Very) low risk**	T1–T2a	< 10	2–6
**Intermediate risk**	T2b-c	10–20	7
**(Very) high risk**	T3a-b	>20	8–10

### mpMRI protocol

mpMRI of the prostate was typically performed on the same day as the planning CT at 1 week prior to the beginning of RT. MRI examinations were performed using a 3 Tesla (38%) or 1.5 Tesla MRI (59%) scanner (Verio, Avanto and Symphony: Siemens Healthcare, Forchheim, Germany), and the signals were acquired using a 32-channel-phased-array-bodycoil (Siemens Healthcare, Forchheim, Germany). Morphological imaging included T2-weighted turbo spin-echo (TSE) sequences in axial and sagittal planes, as well as precontrast T1-weighted TSE sequences in coronal planes covering the prostate and the seminal vesicles. DWIs were acquired using single-shot spin-echo-echo planar imaging with different b-values. Dynamic contrast-enhanced MRI (DCE-MRI) was acquired using 3D T1-weighted spoiled gradient echo sequence and contrast agent was injected using a motorized power injector. The DCE-MRI datasets were transferred to a dedicated radiological workstation (Multimodality Workplace: Siemens Healthcare, Forchheim, Germany) and analyzed by a board-certified radiologist. Three patients (2.5%) underwent an equivalent MRI study in other institutions prior to RT.

### Risk-adapted institutional treatment stratification and target volume definition

Planning CT and mpMRI were usually scheduled on the same day to allow for image fusion and improved MR-based target volume delineation [29,30]. Target volume definition was performed according to current guidelines [7]. For low-risk disease, only the prostate was included in the clinical target volume (CTV). In the intermediate-risk and high-risk groups the CTV was extended with a radial margin of up to 5 mm around the prostatic capsule including the base of the seminal vesicles to account for the increased risk of microscopic ECE. In the case of macroscopic ECE diagnosed on MRI, an additional margin around the extracapsular spread was chosen. Seminal vesicles were included in the CTV when tumor invasion was clinically or radiologically suspected.

RT was planned depending on the risk group. A total dose of 72–76 Gy (2 Gy per fraction, 5 fractions per week) using 3D-conformal RT or dynamic IMRT techniques was delivered. Patients were instructed in the use of a specific bladder and rectum filling protocol to minimize interfraction internal movement of the pelvic organs. ADT was prescribed in the absence of contraindications according to current evidence for 4–6 months for intermediate-risk patients and for 2–3 years for high-risk patients [6,28,31-33]. No ADT was given in the case of low-risk patients.

### Analysis of potential risk factors predicting tumor upstaging and seminal vesicle infiltration using mpMRI

To identify risk factors which increased the probability of tumor upstaging and the detection of seminal vesicle involvement using mpMRI, the following patient characteristics were investigated in univariate and multivariate analyses: age; the Gleason score; serum PSA level; initiation of ADT before MRI; and in the case of seminal vesicle involvement, initial tumor stage. For the analysis, the Gleason score, PSA level and tumor stage were assigned to the low-risk, intermediate-risk and high-risk groups using the cut-off values mentioned above [28].

### Statistical methods

Agreement between ratings using CT and MRI was summarized using Cohen’s weighted kappa coefficient (with squared weights) and the corresponding 95% bootstrapped confidence interval (CI), or as the percentage agreement with the corresponding 95% (Wilson) CI. Logistic regression was used to examine the association between age, PSA level, the Gleason score, tumor stage and anti-hormonal therapy with the probability of upstaging (MRI versus clinical staging). A significance level of 0.05 was used throughout. All analyses were performed in the R programming language (version 3.1.0) [34].

## Results

### Patient characteristics

Patient characteristics are summarized in Table 2. The median age of the 122 study patients was 71.5 +/− 5.9 years with a range of 50.9 to 83.3 years. 42.6% of the patients (n = 52) had a pre-treatment serum PSA level of < 10 ng/ml, 31.1% (n = 38) a PSA level of 10–20 ng/ml, 26.2% (n = 32) presented with a PSA level of > 20 ng/ml. After histopathological grading using ultrasound-guided transrectal biopsy 41% (n = 50) of the patients had a Gleason score of < = 6, 39.3% (n = 48) had a Gleason score of 7 and 18.9% (n = 23) had a Gleason score of 8–10. The Gleason score was not assessable in one patient.

**Table 2:** **Patient characteristics (n = 122)**

**Patient parameter**	**Value**
**Median age (years; range)**	71.5 (50.9–83.3)
**Gleason score, n (%)**	
Low risk (2–6)	50 (41%)
Intermediate risk (7)	48 (39.3%)
High risk (8–10)	23 (18.9%)
**Serum PSA (ng/ml), n (%)**	
Low risk (<10)	52 (42.6%)
Intermediate risk (10–20)	38 (31.1%)
High risk (>20)	32 (26.2%)
**Initial tumor stage (clinical examination and CT), n (%)**	
Low risk (T1–2a)	53 (43.4%)
Intermediate risk (T2b-c)	44 (36.1%)
High risk: extracapsular extension (T3a)	21 (17.2%)
High risk: seminal vesicle infiltration (T3b)	4 (3.3%)

ADT had been initiated before the MRI scan in 46.7% of the patients (n = 57). The median duration of ADT before RT was 51.5 days with a duration of > 6 months in only 3.8% of the patients who had received both ADT and RT (n = 2).

Based on clinical staging before the MRI scan, the UICC clinical stage for prostate cancer was T1c-T2a in 43.4% patients (n = 53), T2b-c in 36.1% of the patients (n = 44) and T3a in 17.2% (n = 21). Seminal vesicle infiltration (T3b) was suspected in 3.3% (n = 4). Additionally, 3.3% of the patients had suspected nodal involvement on the CT scan. According to the criteria of the NCCN 2014 guidelines [28], each T-stage was assigned to the corresponding risk group for further analysis; this resulted in 43.4% of tumors being classified as being in the low-risk group (T1-2a), 36.1% as being in the intermediate-risk group (T2b-c) and 20.5% being in the high-risk group (T3). Taking tumor staging, PSA measurement and the Gleason score together, clinical staging before the MRI scan resulted in a risk group distribution for low, intermediate and high-risk of 11.5%, 42.6% and 43.4%, respectively according the NCCN prostate cancer guidelines. 2.5% had suspected nodal involvement based on CT.

### T stage shift as a result of the mpMRI scan

**Figure 1:**
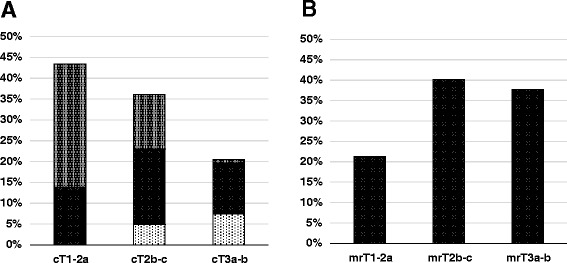
**Distribution of T stage (A) according to CT and DRE and (B) after mpMRI of the prostate. (A)** The relative extent of upstaging (grey) und downstaging (light grey) based on mpMRI are shown compared to unchanged T stage (black) for every initial T stage.

After an mpMRI scan tumor upstaging was observed in 43.4% of the patients (n = 53) relative to the original clinical and CT-based staging. Downstaging as a result of an mpMRI scan occurred in 12.3% (n = 15) in the total population and in 14% (n = 8) of the 57 patients who had received ADT before RT. Tumor stage showed an agreement of 44.3% (95% CI: 0.36, 0.53) between CT and clinical staging versus MRI staging (k = 0.26; 95% CI: 0.11, 0.40). Low-risk T stage tumors (T1-2a; n = 53) were upstaged by MRI in 67.9% of patients, whereas intermediate-risk tumors (T2b-c; n = 44) were upstaged in 36.4% and downstaged in 13.6%. Finally, high-risk tumors (T3a-b; n = 25) were downstaged in 36% of patients, and in a single patient (2.5%) was upstaged to a locally invasive tumor (T4) as a result of mpMRI (Table 3). Depending on the initial risk factors such as serum PSA level and the Gleason score, tumor upstaging in T1-T2 tumors (n = 97) was observed in all subgroups, ranging from 45.2% (low-risk Gleason score < 6) to 73.9% (high-risk PSA level > 20 ng/ml; Table 4 and Figure 1).

**Table 3:** **Distribution of prostate cancer T stage before and after mpMRI of the prostate**

	**Initial clinical staging**	**Relative upstaging by mpMRI**	**Relative downstaging by mpMRI**	**mpMRI staging**
**T1–T2a ((very) low-risk)**	53 (43.4%)	67.9%	n.a.	31 (19.4%)
**T2b-c (intermediate-risk)**	44 (36.1%)	36.4%	13.6%	49 (40.6%)
**T3a-b ((very) high-risk)**	25 (20.5%)	2.5%	36%	46 (37.7%)

T stage for the patients (n = 122) was attributed to specific risk groups according to the National Comprehensive Cancer Network prostate cancer guidelines. Initial clinical staging included digital rectal examination, ultrasound-guided transrectal biopsy as well as an abdominal and pelvic contrast-enhanced CT scan.

**Table 4:** **Incidence of prostate cancer upstaging regarding T stage after mpMRI**

**cT1–2 Tumors**	**n**	**Tumor upstaging (n)**	**Percentage of tumor upstaging**
Gleason score < 7	42	19	45.2%
Gleason score 7	40	24	60%
Gleason score > 7	15	8	53%
PSA level < 10 ng/ml	44	22	50%
PSA level 10–20 ng/ml	30	16	53.3%
PSA level > 20 ng/ml	23	17	73.9%

Incidence of prostate cancer upstaging regarding T stage after mpMRI depending on initial serum PSA level and the Gleason score in patients initially staged as cT1–cT2 (n = 97).

### Changes in risk group distribution as a result of the mpMRI scan

**Figure 2:**
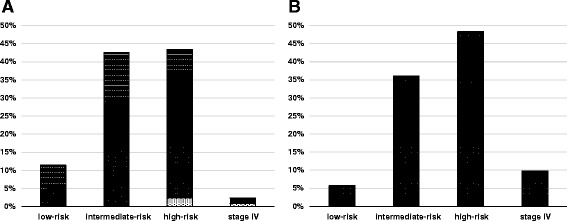
**Distribution of overall risk groups based on PSA, Gleason score and (A) T stage determined by CT and DRE compared to (B) T stage assessed by mpMRI. (A)** The relative extent of risk group upshift (grey) und downshift (light grey) due to mpMRI-based T stage changes are shown for every initial risk group compared to patients without risk group shift (black).

Overall, mpMRI led to an upward shift of the risk group in 25.4% patients and to a downward shift in 3.3% when compared with the initial risk group definition based on serum PSA, Gleason score as well as the initial T stage determined by DRE, ultrasound-guided biopsy and CT. Depending on the initial risk group, an upward shift after mpMRI occurred in the low-risk, intermediate-risk and high-risk group in 57.1%, 30.8% and 13.2%, respectively. A downward shift occurred only in the initial high-risk group in 5.7%. The risk group showed an agreement rate of 71.3% (95% CI: 0.63, 0.79) between clinical staging and MRI staging (*k* = 0.57; 95% CI: 0.44, 0.72). While the percentage of patients assigned to low-risk and intermediate-risk groups decreased, there was a substantial increase observed in the percentage of patients assigned to the high-risk group; there was also a considerable increase in the percentage of patients identified as having stage IV disease with nodal or distant metastases. The resulting risk group distribution before and after mpMRI is demonstrated in Table 5 and Figure 2.

**Table 5:** **Risk group distribution before and after mpMRI of the prostate**

	**Clinical staging**	**Relative upward shift by mpMRI**	**Relative downward shift by mpMRI**	**mpMRI staging**
**Low-risk group**	14 (11.5%)	57.1%	n.a.	7 (5.7%)
**Intermediate-risk group**	52 (42.6%)	30.8%	0%	44 (36.1%)
**High-risk group**	53 (43.4%)	13.2%	5.7%	59 (48.4%)
**Nodal metastases (cN1)**	3 (2.5%)	0%	33.3%	9 (7.4%)
**Distant metastases (cM1)**	0	n.a.	n.a.	3 (2.5%)

Risk groups for patients (n = 122) were defined according to the National Comprehensive Cancer Network prostate cancer guidelines based on T stage, serum PSA level and the Gleason score derived from prostate biopsy. n.a. = not available.

### Therapeutic implications of the tumor stage / risk group shift as a result of the mpMRI scan

Changes in target volume definition as a result of MRI tumor stage occurred in 30.3% of all patients (n = 37) according to our institutional guidelines. Among the 14 patients who presented with low-risk features, 57.1% (n = 8) were shifted to a higher risk group because of MR-based tumor upstaging. Consequently, the CTV was extended beyond the prostatic capsule to account for the increased risk of microscopic ECE and/or SVI. In the 52 patients in the intermediate-risk group, 25% (n = 13) were shifted to the high-risk group because of tumor upstaging, resulting likewise in an increased periprostatic target volume margin. Finally, in the 53 patients initially classified as being in the high-risk group 5.7% were downstaged using MRI (n = 3) with a consequent reduction in the target volume margin and another 5.7% had newly diagnosed SVI resulting in a target volume extension to the whole seminal vesicles. Additionally, nodal or distant metastases were diagnosed in 5.8% (n = 3) intermediate-risk patients and 13.2% high-risk patients (n = 7) resulting in a change in treatment from locally confined RT of the prostate to systemic therapy and/or pelvic irradiation.

Overall, the recommendations regarding the duration of ADT were changed in 29.5% of the patients (n = 36) after mpMRI of the prostate. Of 14 patients initially assigned to the low-risk group, 42.9% (n = 6) were shifted to the intermediate-risk group requiring short-term ADT (4–6 months) and 14.3% (n = 2) were shifted to the high-risk group with a consequent recommendation for long-term ADT (2–3 years). Among the intermediate risk group (n = 52), 25% (n = 13) were shifted upwards to the high-risk group resulting in a prolonged ADT duration, and 5.8% (n = 3) had newly diagnosed stage IV disease with an indication for palliative ADT. Finally, in the 53 patients presenting with high-risk features before MRI, stage IV disease was diagnosed using MRI in 13.2% (n = 7) requiring palliative ADT. 3.8% of the patients (n = 2) were shifted downwards to intermediate-risk disease with an indication for short-term ADT, and one patient (1.9%) was shifted downwards to the low-risk group requiring no ADT.

### Risk factors for tumor stage upstaging using mpMRI

Potential risk factors which could increase the probability of tumor upstaging as a result of the mpMRI scan of the prostate were analyzed in patients initially staged as T1–2 (n = 97) using univariate and multivariate logistic regression including PSA level, the Gleason score, age and neoadjuvant ADT. PSA level, the Gleason score and T-stage were categorized according to the NCCN guidelines into low, intermediate and high-risk groups [28]. Low-risk and intermediate-risk features were analyzed separately as a relative risk factor as compared with high-risk features. Patients presenting with intermediate-risk PSA levels had a significantly smaller probability of being upstaged as a result of MRI than patients in the high-risk PSA group (p = 0.029); this probability remained significant in multivariate analysis (p = 0.043). This trend was also observed for lower PSA levels (<10 ng/ml), but did not reach statistical significance in univariate (p = 0.064) and multivariate analysis (p = 0.096). None of the other variables investigated were significantly associated with MR-based tumor upstaging (Table 6). Of note, downstaging of the T-stage among patients initially staged as T3a-b was not associated with any of the variables considered in our analysis (data not shown).

**Table 6:** **Multivariate logistic regression analysis of potential risk factors for T upstaging as a result of mpMRI**

	**OR**	**95% confidence interval**	**p-value**
**Low-risk PSA (<10 ng/ml)**	0.37	(0.11, 1.15)	0.096
**Intermediate-risk PSA (10–20 ng/ml)**	0.29	(0.08, 0.93)	0.043
**Low-risk Gleason score (<7)**	0.99	(0.27, 3.65)	0.99
**Intermediate-risk Gleason score (7)**	1.88	(0.51, 7.11)	0.34
**Age**	1.02	(0.95, 1.10)	0.54
**ADT pre-mpMRI**	1.34	(0.55, 3.25)	0.52

Multivariate logistic regression analysis was used to demonstrate the relative probability of tumor upstaging after mpMRI of the prostate in patients initially staged as cT1-cT2 (n = 97) depending on specific patient parameters. Patients in the intermediate-risk PSA group had a significantly lower probability of upstaging due to mpMRI than patients in the high-risk PSA group (p = 0.043). OR = Odds ratio. ADT = Androgen deprivation therapy.

### Risk factors for seminal vesicle involvement detected using mpMRI

Potential risk factors increasing the probability of newly diagnosed seminal vesicle infiltration using mpMRI were analyzed by means of univariate and multivariate logistic regression in all patients (n = 122) including PSA level, the Gleason score, initial tumor stage, age and neoadjuvant ADT. PSA level, the Gleason score and T-stage were divided according to the NCCN guidelines into low, intermediate and high-risk groups. High-risk and intermediate-risk features were analyzed separately as a relative risk factor as compared with low-risk features. PSA levels above the low-risk range were associated with an increased risk which reached statistical significance in univariate analysis for the intermediate-risk PSA range (p = 0.041), but not for the high-risk PSA range (p = 0.11). The same trend was found in multivariate analysis which, however, was not quite statistically significant (p = 0.058). In contrast, none of the other factors that were evaluated appeared to play a significant role in this investigation.

## Discussion

MRI of the prostate has already been established as a valuable tool for radiation oncologists in improving target volume delineation [35-37] and has also been investigated for treatment planning [38]. It has been shown that morphologic MRI enables a more reproducible definition of the prostate gland [39-41], particularly when it comes to the definition of the prostate apex [42,43]; it leads to a smaller CTV volume and the sparing of more normal tissue relative to CT-based target volume definition [44]. Consequently, in prostate cancer it has been suggested that MRI-based treatment may reduce treatment-associated toxicity [30].

Apart from precise organ delineation, the accurate radiological tumor staging of prostate cancer is playing an increasingly important role in the era of IMRT [8]. Using highly-conformal RT techniques and small planning target volume margins, the detection and inclusion of extracapsular disease and seminal vesicle infiltration into the treatment volume is of paramount importance in avoiding a geographic miss and consequent tumor under-dosage [45].

MRI of the prostate can offer additional benefit in RT planning because it is regarded as the most reliable imaging modality regarding the determination of the local extension of prostate cancer, and it exhibits the highest congruence with the “gold standard” of post-operative pathological staging [10,11].

Surgical series have demonstrated that both the digital rectal examination, as well as other radiological techniques such as CT scans of the abdomen and transrectal ultrasound, only exhibit low correlations with definitive pathological staging after radical prostatectomy; indeed, they only accurately predict the extent of the local tumor 61–70% of the time [19,20]. In contrast, morphological T1- and T2-weighted MR imaging alone has already shown an accuracy of 84% for the detection of extraprostatic disease in clinically staged T2 prostate cancer [46]. In recent years, mpMRI sequences have been introduced into prostate cancer imaging in order to further enhance tumor staging [14]. As a result of the early rapid enhancement and early washout of prostate cancer tumors, DCE-MRI provides a means of determining tumor extension more accurately than morphological imaging [10], and has demonstrated an improved overall staging accuracy of 95% in a single-institution series [17]. Interestingly, this improvement in prostate cancer tumor staging due to DCE-MRI has been found to be particularly important for less experienced readers [47]. Likewise, recent literature reviews have confirmed that additional DWI can detect prostate cancer tumor foci more accurately than conventional anatomic imaging alone [12,48]. For these reasons, mpMRI of the prostate has been established in our institution as a standard imaging procedure in all patients before primary RT for prostate cancer. Although this approach is not uncommon among RT units, it cannot at the moment be considered as a modality with interdisciplinary consensus. Current guidelines recommend the use of mpMRI for local staging of prostate cancer ahead of curative therapy mainly for high-risk patients [28,31], whereas its value is questioned in patients presenting with low-risk features [49]. The purpose of the present study was therefore to analyze retrospectively the extent of the shift in tumor stage as a result of the use of mpMRI, and the consequent impact on risk group distribution and treatment decisions in primary RT for prostate cancer.

In our patient series, a considerable impact on prostate tumor staging as a consequence of the use of mpMRI was observed, with tumor upstaging in 43.4% and downstaging in 12.3% of patients. These findings are consistent with the findings from a previously published series of 199 patients with localized prostate cancer; in this series morphological MRI scans of the prostate, without DCE imaging or DWI, led to tumor upstaging in 52% and to tumor downstaging in 3% of patients before RT [25].

Although our study showed a trend towards a significantly lower probability of MRI-based tumor upstaging in patients with an intermediate or low-risk PSA level, there was no subgroup identifiable where it appeared reasonable to omit mpMRI before RT. Even in the low-risk subgroups (Gleason score < 7; PSA level < 10 ng/ml), a substantial upstaging of 45–50% could be observed. However, taking all of the established risk factors, such as the Gleason score, PSA level and tumor stage together, mpMRI of the prostate only led to a change in risk group in 25.4% of the patients in our series. This might be explained by the fact that patients with a locally advanced tumor stage detected by MRI may often present with other adverse features which had already been assessed before MRI.

Tumor stage shift and change in risk group taken together resulted in a change in the RT dose and target volume, as well as in the recommendations for additional ADT in 30% of patients. These findings are in concordance with recent data from a study by Chang et al. [24] who reported a significant upstaging of 29% before primary RT for prostate cancer in a comparable patient series, using morphological MRIs without multiparametric dynamic sequences. Interestingly, Clure et al. reported a similar extent of treatment adaptation of 27% as a result of MRI staging in a series of 104 patients who underwent robotic-assisted laparoscopic prostatectomy [50]. Similarly, Hricak et al. reported a change in surgical plan based on MRI scans in 39% of patients who underwent radical prostatectomy for localized prostate cancer [51].

Whereas it seems to be legitimate to increase treatment intensity for patients with mpMRI based upstaging, caution should be exerted for treatment de-intensification in cases where mpMRI led to downstaging: in our series, a considerable number of downstaging was found in patients who received ADT before RT. It is, however, mainly not determinable whether MR-based downstaging occured due to previous diagnostic inaccuracy of the pre-MRI staging or if it was an ADT effect. If the latter can’t be definitely ruled out, downstaging should not subsequently lead to de-intensification of radiotherapy. In order to avoid this uncertainty of ADT interference, mpMRI should be conducted as part of the initial staging procedure before start of any neoadjuvant treatment.

Our study had several limitations. First, all data were reviewed retrospectively and staging information was obtained from patient histories. In addition, the reporting radiologists had not been blinded to previously obtained clinical staging. However, these circumstances may represent the daily practice in a major cancer center more accurately than would be the case in a dedicated prospective trial. Second, clinical and MRI staging were not correlated to oncological outcome as has been reported in other studies [25,52]; this was because the follow-up time was limited as we evaluated a very recent patient series. The main focus of our study was on the impact of MRI staging on treatment decisions, not on its predictive value. mpMRI sequences were not reported separately from morphological MRI, so their additional value for treatment decisions could not be independently assessed. Additionally, in contrast to studies regarding the surgical treatment of prostate cancer, MRI staging could not be validated using definitive pathological staging. Finally, the impact of MRI on target volume delineation and treatment volume relative to CT-based planning was not analyzed in the current study, because it has been investigated extensively by others [29,30,53].

## Conclusions

The use of mpMRI in patients admitted for primary RT for prostate cancer resulted in a substantial shift in tumor stage in > 50%, with consequent treatment adaptations in nearly one third of the patients. In our single center series, we could not identify any subgroup of patients where mpMRI did not lead to considerable upstaging. In conclusion, our findings suggest that patients from all risk groups may benefit from mpMRI of the prostate regarding the choice of the optimal risk-adapted RT.
